# Infrared micro-spectral imaging: distinction of tissue types in axillary lymph node histology

**DOI:** 10.1186/1472-6890-8-8

**Published:** 2008-08-29

**Authors:** Benjamin Bird, Milos Miljkovic, Melissa J Romeo, Jennifer Smith, Nicholas Stone, Michael W George, Max Diem

**Affiliations:** 1 Department of Chemistry and Chemical Biology, Northeastern University, Boston, USA; 2 Biophotonics Research Group, Gloucestershire Hospitals NHS Foundation, Gloucester, UK; 3 School of Chemistry, University of Nottingham, Nottingham, UK

## Abstract

**Background:**

Histopathologic evaluation of surgical specimens is a well established technique for disease identification, and has remained relatively unchanged since its clinical introduction. Although it is essential for clinical investigation, histopathologic identification of tissues remains a time consuming and subjective technique, with unsatisfactory levels of inter- and intra-observer discrepancy. A novel approach for histological recognition is to use Fourier Transform Infrared (FT-IR) micro-spectroscopy. This non-destructive optical technique can provide a rapid measurement of sample biochemistry and identify variations that occur between healthy and diseased tissues. The advantage of this method is that it is objective and provides reproducible diagnosis, independent of fatigue, experience and inter-observer variability.

**Methods:**

We report a method for analysing excised lymph nodes that is based on spectral pathology. In spectral pathology, an unstained (fixed or snap frozen) tissue section is interrogated by a beam of infrared light that samples pixels of 25 μm × 25 μm in size. This beam is rastered over the sample, and up to 100,000 complete infrared spectra are acquired for a given tissue sample. These spectra are subsequently analysed by a diagnostic computer algorithm that is trained by correlating spectral and histopathological features.

**Results:**

We illustrate the ability of infrared micro-spectral imaging, coupled with completely unsupervised methods of multivariate statistical analysis, to accurately reproduce the histological architecture of axillary lymph nodes. By correlating spectral and histopathological features, a diagnostic algorithm was trained that allowed both accurate and rapid classification of benign and malignant tissues composed within different lymph nodes. This approach was successfully applied to both deparaffinised and frozen tissues and indicates that both intra-operative and more conventional surgical specimens can be diagnosed by this technique.

**Conclusion:**

This paper provides strong evidence that automated diagnosis by means of infrared micro-spectral imaging is possible. Recent investigations within the author's laboratory upon lymph nodes have also revealed that cancers from different primary tumours provide distinctly different spectral signatures. Thus poorly differentiated and hard-to-determine cases of metastatic invasion, such as micrometastases, may additionally be identified by this technique. Finally, we differentiate benign and malignant tissues composed within axillary lymph nodes by completely automated methods of spectral analysis.

## Background

At present, breast cancer is the most common malignancy found among women, with high death rates recorded in the United Kingdom (13,000 p.a.) and the United States of America (40,000 p.a.) [1]. The introduction of mammography programs, together with greater public awareness of breast cancer, has significantly improved the early detection of breast cancers and thus their effective treatment. Although x-ray mammography can readily identify areas of tumour growth within the breast, it cannot be reliably used to diagnose whether a tumour is benign or malignant in nature. The accurate diagnosis of a suspicious lesion therefore necessitates an invasive procedure to obtain a tissue biopsy. An additional tool for diagnosis or staging of disease is the assessment of lymph nodes in the ipsilateral axilla. The presence of metastasis is an indicator for local disease recurrence and thus a method for identifying patients that are at high risk of developing disease that could spread throughout the body. The well established procedure to assess lymph node metastasis is axillary lymph node dissection (ALND). This involves the surgical removal of all or most lymph nodes that exist under the arm. However, this is a rather substantial surgical procedure and can lead to several serious side effects, including shoulder dysfunction and lymphodema [2]. More recently, intra-operative diagnosis of excised lymph nodes has been used within a small number of hospitals. Such rapid diagnoses are made upon the sentinel lymph node that has direct lymphatic connection to the breast tumour [3]. Surgical studies have clearly shown that if metastasis cannot be found in the sentinel lymph node, the chance of disease being found further down the chain of nodes is negligible, thus alleviating the necessity to remove all nodes present [3].

Biopsy material collected during these surgical procedures are subsequently scrutinised using traditional histological techniques [4], whereby dyes are introduced that stain different cellular components different colours. These staining patterns provide the basis for morphological pattern recognition, allowing a trained observer to distinguish between healthy and diseased tissue. However, traditional histology remains a subjective technique, with significant problems often encountered. These include missed lesions and unsatisfactory levels of inter- and intra-observer agreement [5-10]. Alternative techniques have been employed to facilitate faster intra-operative diagnosis of sentinel nodes, including imprint cytology [11,12], and frozen section analysis [13,14]. The processing of samples is accelerated for these techniques, involving an analysis time of approximately 30–60 minutes. Yet, both approaches report wide variation in their sensitivity to detect cancerous lesions, detection levels as low as 44% and as high as 93% when compared with conventional histology [11-17]. These variations indicate that such methodologies do not solve the problems associated with screening lymph nodes.

The lack of a reliable tool to swiftly diagnose both conventional and intra-operative surgical specimens has led to a considerable amount of interest in the application of a spectroscopic approach. Fourier Transform infrared (FT-IR) spectroscopic imaging is rapidly becoming a key technique for biomedical spectroscopy since it provides spatially resolved chemical characterisation of microscopic areas. Using this technique, contrast between different spatial areas occurs due to inherent chemical differences found within cells of the tissue, producing molecule-specific vibrational signatures. A chemical image of the tissue section can then be constructed that is similar to the morphological interpretation of a stained image, thus enabling the identification of tissue classes and providing an insight into their molecular composition. An FT-IR spectroscopic approach, therefore, has several advantages over conventional histology. For example, an infrared micro-spectral image collected from a tissue section measuring 5 mm × 5 mm, using a spatial resolution of 25 μm × 25 μm per spectral measurement, consists of 40,000 individual objective measurements that describe the biochemistry of the tissue regions. Recent advances in instrumentation, that employ multi-channel detector systems and fast interferometry, allow the collection of such spectral datasets within a matter of minutes, and the continuing improvements in technology are expected to reduce data collection times dramatically. Paraffin embedded specimens that have been deparaffinised, or snap frozen sections may be examined using this non-destructive technique. The most important aspect of the proposed FT-IR approach is that it provides an unbiased computer based technique that can ultimately be automated. This paper reports progress made within our laboratories to automatically diagnose spectral data collected from both frozen and deparaffinised axillary lymph node tissues.

## Methods

**Figure 1:**
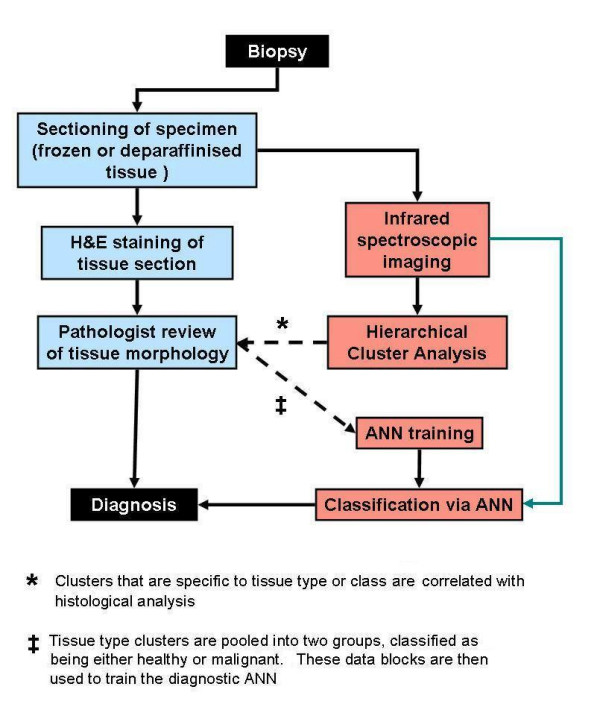
**Work flow diagram for training and test phases of diagnostic algorithm**. The current protocol for cancer diagnosis and grading of biopsy material involves the sectioning of samples, H&E staining, and an assessment of tissue and cellular morphology by a pathologist (left, blue shaded boxes). To implement an automated protocol of analysis using infrared micro-spectral imaging, a training phase is required to develop a robust diagnostic algorithm (right, red shaded boxes). The final paradigm for automated analysis involves infrared micro-spectral imaging of unstained tissue, followed by computer analysis using the diagnostic algorithm (right, green arrow). A two step approach for training a neural net was employed in this investigation. Spectral data sets recorded from tissues are initially scrutinised via hierarchical cluster analysis (HCA), a completely unsupervised method of analysis, to produce groups of infrared spectra that are specific to tissue type or class. This is achieved by directly correlating spectral images constructed from HCA analysis, to morphological interpretations that were made by a pathologist using the stained tissue. These tissue specific groups of spectra were then pooled into two separate data blocks, classed as being either healthy or malignant in nature. These newly compiled data blocks were then used to train a diagnostic ANN.

Figure 1 shows a schematic diagram of the work flow for training/validation and test phases of the work reported here. It should be noted that the time-consuming cluster analysis is required only in the training phase of the diagnostic algorithm, and that the final analysis of unknown lymph node data sets can be performed within a minute of data collection.

### Specimen collection and sample preparation

This manuscript presents collaborative research undertaken at three different spectroscopic laboratories. As a consequence, adjustments to the type of sample preparation and mode of data acquisition were made to allow additional spectroscopic measurements via Raman microscopy [18], and the analysis of archival tissue blocks. Tissue specimens investigated in the UK involved the collection of additional biopsy samples, during routine surgical investigations, with full written consent of the patient. Ethical approval for these studies was provided by the Gloucestershire Local Research Ethics Committee and Gloucestershire Royal Hospital. These samples were immediately mounted on acetate paper, placed in a 2 mL cryovial, and snap frozen in liquid nitrogen. Samples were then cut with a freezing microtome to provide tissue sections of 6 μm thickness, which were mounted on barium fluoride substrates to enable acquisition of spectra in transmission mode. This methodology was adopted since it minimised the contamination of the sample from fixing and mounting agents, negated possible changes to sample biochemistry via conventional paraffinisation and deparaffinisation procedures, and allowed additional Raman spectral data to be collected without the background affects associated with glass substrates. This manuscript shall not detail the results from the Raman investigations since these are already partially documented in a previous work and were undertaken with the goal of collecting a library of data for the development of an *in vivo *optical tool [18].

Tissue specimens investigated in the USA were cut from the archival tissue banks held at Cook County Hospital, Chicago, IL. The use of such a bank allowed the acquisition of a greater number of tissues with a firm histological diagnosis. These specimens were stored as paraffin-embedded tissue blocks and were cut, using a microtome, to provide tissue sections of 6 μm thickness and subsequently deparaffinised. These sections were mounted on reflective substrates that allow spectra to be recorded in transflection mode [19]. In this measurement mode, the probing IR beam passes the sample, is reflected by the substrate, and passes the sample again.

The investigation of tissue that has been chemically treated is unavoidable in this scenario, and is likely to have small affects upon the biochemistry of the tissue. For example, during the wax embedding procedure, tissue sections are subject to a series of solvents with decreasing polarity (water, ethanol/water, xylene, paraffin). This can dissolve lipids and consequently remove them from the tissue sections. Therefore the application of this sample preparation would not be recommended for the investigation of tissue morphology inside adipose rich tissues. In addition, the complete deparaffinisation of tissue is recommended for spectroscopic analyses since paraffin exhibits strong bands in the methyl and methylene stretching (3000 – 2800 cm^-1^) and deformation (~1450 cm^-1^) regions of the infrared spectrum, which may confound subsequent multivariate analyses. Despite these chemical treatments, potent biochemical information is retained as highlighted by conventional immunohistochemical studies. For example, IR spectroscopic investigations on frozen [20] and de-paraffinised [21] brain tissues excised from rats, displayed similar sensitivities in their ability to identify anatomical features of both healthy and diseased tissues.

The use of reflective substrates (Kevley Technologies, Chesterland, Ohio, USA) for the investigation of these tissues was a decision based upon cost, and reflected the need for cheap consumable IR substrates that could be used in a clinical environment (~US$ 1 per slide compared with > US$200 for BaF_2 _or CaF_2_). These slides are made of glass coated with a thin Ag/SnO_2 _layer. They are chemically inert and nearly transparent to visible light. However, they reflect mid-infrared radiation almost completely and are therefore ideal and inexpensive substrates for infrared micro-spectroscopy in reflection mode, as they allow both visual and infrared images to be collected from the same sample.

As part of this study we have examined more than 1.4 million infrared spectra recorded from 30 whole excised lymph nodes (10 by frozen sectioning and 20 by deparaffinisation of wax-embedded tissues). We report here a subset of this spectral library, containing c.a. 240,000 infrared spectra recorded from 6 excised lymph nodes (3 by frozen sectioning and 3 by deparaffinisation of wax embedded tissues).

### Spectroscopic data acquisition and processing

Spectroscopic imaging data were acquired using a commercially available infrared spectrometer (Perkin-Elmer, Spectrum One) coupled to an infrared microscope (Perkin-Elmer, Spectrum Spotlight 300). This instrument employs a sensitive mercury-cadmium-telluride (MCT) linear array detector system (16 elements), coupled with a computerised stage, to collect large spectroscopic images from a sample. In this study, spectroscopic images were recorded from entire lymph nodes and could vary in size from c.a. 5 mm^2 ^to 10 mm^2^. Each pixel sampled a 25 μm × 25 μm area at the sample plane, providing images that contained between 40,000 and 160,000 individual infrared spectra. Spectral data were acquired either in transmission mode for frozen tissues or transflection mode for deparaffinised tissues. All spectral measurements were recorded using a mirror speed of 1 cm s^-1^, a spectral resolution of 8 cm^-1^, and a minimum signal-to-noise ratio of 200 (signal: maximum of the amide I band; noise: the standard deviation in the spectral range 1800 – 1900 cm^-1^). Each spectrum was fast Fourier transformed using Norton-Beer apodisation to yield single beam spectra. An appropriate background spectrum was collected outside the sample area to ratio against the single beam spectra. The resulting ratioed spectra were then converted to absorbance. The acquisition of spectroscopic images of this magnitude was quite time-consuming, and could require several hours for very large tissue sections. However, more recent instrumentation that utilise Focal Plane Array (FPA) detector technology provide far superior rates of data acquisition at higher spatial resolution. Thus images of similar magnitude can be collected in ca. 10 minutes. A more detailed account of these instruments shall be included later in this manuscript. Following spectral data acquisition, samples were either directly stained (deparaffinised tissue), or adjacent sections cut and stained (frozen tissue) using standard H&E protocols. This allowed direct comparisons to be made between pseudo colour maps constructed from unsupervised methods of spectral analysis and traditional histopathology.

As reported in previous contributions [22,23], infrared spectra that display very small absorbance values and are collected from the edges of a sample, where the tissue is thin or does not adhere well to the substrate, can in a some cases be contaminated with artefacts associated with dispersion. This is an optical effect where spectra can become distorted by the superposition of a dispersive line shape during Fourier transformation, and is mostly prevalent in measurements that are acquired in transflection mode. A few techniques have been suggested to process and correct the contaminated spectral data. These include methods of spectral un-mixing [24], or performing a secondary phase correction on the spectra [22]. Since these data processing techniques are computationally intensive, we have adopted a method that removes all data with such artefacts since the number of bad pixels in an image is still very small (< 1%). By application of a stringent signal to noise test (as detailed below), the overwhelming majority of spurious data is removed since they commonly display features of poor signal intensity. The remaining bad pixels are identified during unsupervised multivariate analysis by means of Hierarchical Cluster Analysis (HCA). During this type of analysis, which is described below, spectra with dispersive line shapes are commonly grouped together into single clusters. Therefore, any remaining spectra with unwanted artefacts can be quickly identified and subsequently removed from any data sets that would be used to train supervised algorithms to classify spectral data.

All spectral data processing and image assembly was performed using the CytoSpec software package , which enables spectral processing and multivariate analysis to be carried out on an entire spectral imaging data set, or "spectral hypercube". Initially, a spectral quality test was performed to remove all spectra recorded from areas where no tissue existed, or displayed poor signal to noise. This was accomplished by subjecting all spectra to a "thickness" test, using settings of 20 and 500 for the minimal and maximal integrated intensity criterion in the wavenumber range of 1600 – 1700 cm^-1^. All spectra that pass the test were then converted to second derivative spectra (Savitzy-Golay algorithm, 9 smoothing points) and vector normalised across the full wavenumber region recorded (4000 – 750 cm^-1^). The former of these procedures produces better resolved peaks and eliminates background slopes, whereas the latter reduces the influence of intensity changes caused by differences in cellular density and thickness of the tissue. Finally, the dimensionality of the spectral hypercube was reduced to only include intensity values recorded in the spectral ranges 3100 – 2800 cm^-1 ^and 1800 – 900 cm^-1^. The C-H stretching region (3100 – 2800 cm^-1^) was included in the analysis because it produced superior classification of tissue types and disease than the biological fingerprint region (1800 – 900 cm^-1^) alone. These fully processed spectral hypercubes were then used for subsequent Hierarchical Cluster Analysis (HCA) and Artificial Neural Network (ANN) analysis.

### Unsupervised methods of tissue classification via HCA spectroscopic imaging

Data within a spectral hypercube are partitioned into classes that reproduce tissue histology by use of Hierarchical Cluster Analysis (HCA). This is a common technique employed for pattern recognition and is completely unsupervised [25]. The aim of the clustering process is to group a given set of unlabelled data into a number of clusters, so that data held within the same group are as similar as possible, and data held within different groups are as dissimilar as possible. The algorithm of this technique can be described in the following manner: First, a distance matrix between all spectra contained within the hypercube is calculated. This matrix contains the complete set of inter-spectral distances (measures of similarity), is symmetric along its diagonal, and has the dimensions *n *× *n*, where *n *is the number of spectra. The two objects (spectra) that are closest to one another (most similar) are merged into a new object (cluster). Thus, the dimension of the distance matrix is reduced to (n - 1) × (n - 1). Subsequently, the distances of the new formed object to all remaining objects is recalculated, and again the two most similar objects are merged. This clustering process is iterated until all objects have been merged into a few clusters. This merging process can be visualised in a tree-like "dendrogram" that can be truncated at different points to reveal different clustering structures. The clusters created during the analysis should contain spectra from histological regions that display comparable spectral characteristics. In contrast, spectra contained in different clusters should exhibit spectral features characteristic of different tissue types. Pseudo-colour "cluster images" can thus be assembled and compared directly with H&E images captured from the same sample. By assigning each cluster a colour, these colours can then be plotted as pixels at the x, y coordinates from which the spectrum was collected. Therefore, pixels with the same colour in the image are spectra that were grouped together into the same cluster. Subsequent to HCA analysis of a spectral hypercube, pseudo-colour images of between 2 and 15 clusters, which describe different clustering structures, were assembled by cutting the calculated dendrogram at different levels. These cluster images were then provided to the collaborating pathologists, who confirmed the clustering structure that best replicated the morphological interpretations they made using the H&E stained tissue section. After these correlations were made, mean average spectra were calculated for each cluster, and those that described artefacts from dispersion were subsequently removed from any further supervised analysis. The spectral hypercubes reported in this manuscript were devoid of clusters that contained such artefacts.

### Pattern recognition by use of artificial neural networks (ANN)

As shall be reported later in this paper, HCA spectroscopic imaging can provide pseudo colour cluster images that are directly comparable to conventional histology. However, the application of this unsupervised technique to classify data sets from multiple lymph nodes is complex and distinctly time prohibitive. For example, a spectral data set collected from a 5 mm × 5 mm section of tissue, with 25 μm × 25 μm pixels, contains 40,000 individual infrared spectra, which may exceed 300 MB of memory. The correlation matrix calculated for this data set, which is used for subsequent clustering, would exceed 4 GB of RAM and requires a 64 bit processor with large memory access. Such an analysis would take several hours, a timescale insufficient for rapid diagnosis. A more practical method to rapidly classify or diagnose recorded spectral data sets would be to use a supervised method of analysis. Several different types of supervised analysis have been employed to classify spectral data from cells or tissues, including linear discriminant analysis (LDA) [26-28], metric bayesian classification [29-31], support vector machines (SVM) [32,33] and artificial neural networks (ANN) [34-37]. In this investigation, neural networks were employed to classify recorded spectral data, since data sets were easily transferable between data acquisition, multivariate analysis, and classification software. ANNs are modelled upon biological nervous systems, such as the brain, to process information and extrapolate common patterns that can be used for classification. The internal mechanics of a neural net and its possible applications are well documented and discussed in detail elsewhere [38]. Within our laboratory we have adopted a two step approach for training a diagnostic neural net. Spectral data sets recorded from tissues are initially scrutinised by HCA to produce clusters that are specific to tissue type or class. These tissue specific clusters of spectra, along with their histopathological diagnosis, are then used to train an artificial neural network that may subsequently serve as a supervised method of analysis. Such a neural net can allow the classification of a large spectral data set, as described above, within one minute. A schematic describing the methodology employed is displayed in Figure 1.

In this study, artificial neural network classification and feature selection was performed using NeuroDeveloper 2.5 (Synthon GmbH, Heidelberg, Germany). For each tissue preparation method (frozen or de-paraffinised), a separate neural net was developed. A single tissue section in each case was utilised for neural net training, and the classifier developed blind tested upon supplementary lymph nodes. We additionally adopted a simple classification scheme, where tissue spectra were classed as being either healthy or malignant in nature. Therefore a lymph node that displayed both histological features was used for training. After HCA spectroscopic imaging was performed on the training lymph node, a fixed number of 150 spectra were randomly extracted from each tissue type cluster. This fixed input number of spectra equated to one tenth of the total spectra contained within the smallest tissue type cluster (capsule tissue of lymph nodes) and helped to avoid overrepresentation of a single tissue type. Spectral data was then pooled into two separate libraries of healthy or malignant tissue. Using these newly assembled, un-biased data sets, two data blocks were constructed for subsequent training and validation of the neural net (split in an 80% to 20% ratio, respectively). The training data block was used to help establish the network parameters that would provide the best possible classification. The validation data block was alternatively used to optimise the generalisation performance of a network that was in training. A final data block, constructed from the tissue spectra that remained in the original tissue type clusters, was used as a final testing set to confirm that a given network had sufficiently broad generalisation power to serve reliably as a diagnostic algorithm.

Before a neural net was actively trained for classification purposes, we applied a spectral feature selection algorithm to the training block of data. This algorithm calculated the covariance of all spectral data points within the training data block for each class type, whether healthy or malignant. A ranking list of covariance values was then assembled for each class type. From this list, the top 120 data points in each class, which displayed a minimum covariance of 95%, were made available for neural net training. This procedure reduced the complexity and dimensionality of the input data and substantially improved the quality and robustness of the classification model. Three-layer, feed-forward networks with 5–120 input neurons, 4–20 hidden units, and 2 output nodes were tested. Resilient back-propagation (Rprop) [39,40] was used as the learning algorithm. Tested Rprop parameters were in the following range: Δ_0 _= 0.075–0.1, Δ_max _= 30–50 and α = 4–5 where Δ_0 _is the initial network update value, Δ_max _is the maximum update value and α is the weight decay term. The training process was stopped when errors of training and validation data sets converged.

## Results and discussion

**Figure 2:**
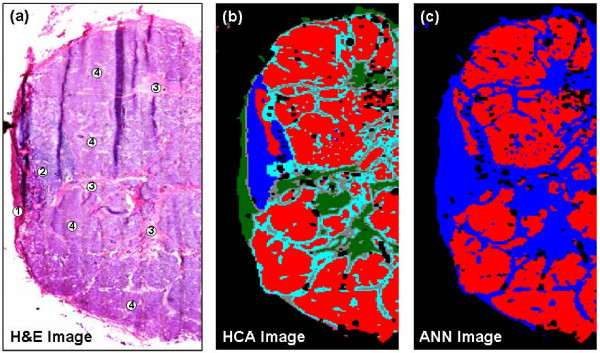
**Frozen tissue analysis using HCA spectroscopic imaging & supervised pattern recognition**. (a) H&E stained image of a positive lymph node PN1. The positive node comprises large regions of cancerous breast tissue (4), remnants of healthy cortex tissue (2), and collagenous tissues such as the capsule (1) and medullary sinuses (3), respectively. (b) HCA spectroscopic image of positive lymph node PN1. The IR imaged area (2.8 mm × 7.5 mm) was mapped using a step size and aperture of 25 μm for a total 33,600 individual IR spectra. The red colour in the image describes areas of cancerous invasion, whereas the blue colour depicts a region of remnant healthy cortex tissue. Green, grey and cyan colours in the image represent regions of collagen containing tissues such as the capsule, medullary cords and sinuses, respectively. (c) ANN image of positive lymph node PN1. The red colour in the image describes regions classified as cancerous by the analysis. In contrast, the blue colour depicts the tissue that was correctly classified as non-cancerous. Black pixels within the image describe spectra that were not able to be classified by the neural net.

The results presented in Figure 2 clearly illustrate the capability of spectroscopic imaging to accurately reproduce tissue pathology. The H&E stained image displayed in Figure 2a was collected from a tissue section cut from a frozen axillary lymph node (labelled as PN1). This tissue section was cut adjacent to the section used for spectroscopic data acquisition, and provides a means to directly compare images constructed from HCA analysis with conventional histology. After routine analysis by a histopathologist, this lymph node was diagnosed as being positive for cancer, since it displayed large regions of cancerous breast tissue invasion and only a small pocket of remnant healthy cortex tissue. The lymph node also displayed typical anatomical structures that contain fibrocollagenous tissues, such as the capsule, medullary cords and medullary sinuses. Since this tissue section displayed all types of tissue commonly found within an excised axillary lymph node, it was chosen to provide a database of reference spectra from which a diagnostic neural net could be trained. Figure 2b displays a typical pseudo colour image that can be constructed using HCA spectroscopic imaging. By subjecting the recorded spectral data set to HCA analysis, a clustering dendrogram that describes the merging process of similar spectra was produced. This was subsequently used for cluster related imaging, whereby multiple images were constructed that reflected different clustering structures. The image displayed in Figure 2b represents the cutting of the dendrogram to reveal a 5-cluster structure. It is clear from Figure 2b that this clustering structure accurately reproduces the histological features of the excised lymph node. Cancerous breast tissue is represented by the red cluster of spectra within the image, whereas the remnant healthy cortex tissue is characterised by the dark blue cluster of spectra. Fibrocollagenous tissues such as the capsule, medullary cords, and medullary sinuses, are represented by the dark green, cyan and grey colours within the image respectively. The colour scheme utilised by this image is entirely arbitrary and does not permit the direct comparison of morphological features with the same colour in different spectroscopic maps. Spectral differences between clusters, which reflect variations in the biochemical composition of different tissue types, can be assessed by calculating and comparing mean cluster spectra. It is far beyond the scope of this paper to provide a detailed account of the spectral differences that were identified among these often diverse tissue types. However, as reported in earlier contributions that examined and diagnosed cancers composed within cervical [41], colon [42], prostate [43], lymph node [23,44] and thyroid tissues [44], spectra collected from diseased or abnormal cells appear to exhibit subtle but distinct changes to the shape, intensity, and ratio, of protein and nucleic acid specific molecular vibrations. This would prove to highlight a significant change in both the protein and nucleic acid composition within these regions.

After verifying the clustering structure that most directly reproduced tissue histopathology, spectra from each cluster were extracted into a reference library, as described previously, and used to train a diagnostic neural net for the classification of frozen axillary lymph node tissues. The trained neural net was then directly applied to the original spectral data set to confirm its sensitivity. Figure 2c displays the classification or ANN image that was constructed after supervised spectral analysis. By analysing the spectral data set using the ANN, each spectrum was classified as being cancerous, healthy or un-identifiable. These three class types were then assigned an individual colour, so that cancerous spectra were coloured red, non-cancerous spectra were coloured blue, and spectra that could not be identified or were rejected were coloured black. These colours were then plotted at the x, y co-ordinates from which each spectrum was recorded, thus creating a pseudo-colour ANN image. The entire classification and image re-construction procedure was completed in approximately one minute. By direct comparison of this ANN image, and those acquired from staining (Figure 2a) and HCA spectroscopic imaging (Figure 2b), a remarkable agreement is observed. Regions of the tissue section that were previously identified as being healthy cortex, capsule, medullary cords or medullary sinuses were correctly classified by the algorithm as being non-cancerous in nature. In contrast, the invading cancerous breast tissue that comprises a majority of the tissue area is correctly classified as being malignant. The number of additional black pixels is also very small, which indicates only a small amount of spectra could not be classified by the algorithm.

**Figure 3:**
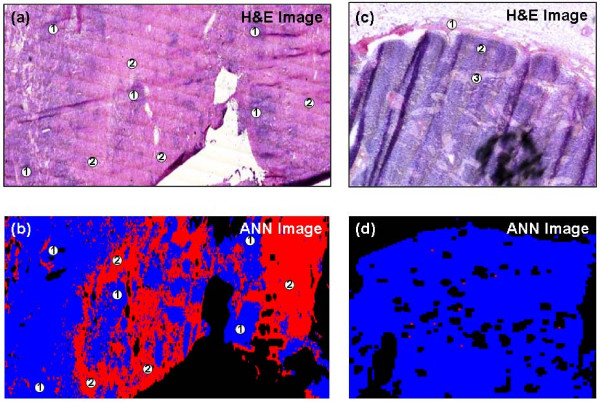
**Frozen tissue analysis using supervised pattern recognition**. (a) H&E stained image of positive lymph node PN2. The IR imaged area (11.2 mm × 3.1 mm) was mapped using a step size and aperture of 25 μm for a total 55,552 individual IR spectra. The positive node comprises large regions of cancerous breast tissue (2) and remnants of healthy nodal tissue (1). (b) ANN image of positive lymph node PN2. The red and blue colours represent the correctly classified cancerous (2) and healthy nodal tissues (1) respectively. Black pixels within the image describe spectra that were not identifiable by the neural net. (c) H&E stained image of negative lymph node NN1. The IR imaged area (2.8 mm × 2.5 mm) was mapped using a step size and aperture of 25 μm for a total 11,000 individual IR spectra. The negative node comprises typical anatomical features of a healthy node. These include the surrounding capsule (1), primary follicles (2) and medullary sinuses (3). (d) ANN image of negative node NN2. The blue colour is representative of healthy nodal tissue and thus correctly classifies the tissue section. The red colour describes the very small number of pixels incorrectly classified as cancerous node by the analysis. Black pixels within the image describe spectra that were not able to be classified by the neural net.

This initial test of the trained neural net proved to be very promising. However, a more demanding and rigorous test would be to apply the same algorithm to spectral data sets collected from different lymph nodes that were not used to train the neural net. Therefore, two additional lymph node data sets were analyzed by the same neural net. The results from these experiments are shown in Figure 3. The H&E stained image displayed in Figure 3a was captured from a frozen section cut from another positive lymph node (labelled as PN2). Within this section, large regions of invading cancerous breast tissue were clearly evident, and only small pockets of remnant non-cancerous tissue remained. As previously described, spectral data were acquired from a directly parallel unstained section, and the recorded data set analysed by the trained neural net. The resulting ANN image, displayed in Figure 3b, again bears a remarkable resemblance to the corresponding stained image. Despite small structural differences due to tissue folding and tearing, there is almost a one-to-one correlation between histology and the spectral diagnosis. The H&E image displayed in Figure 3c was alternatively captured from a frozen section cut from a negative or healthy lymph node (labelled as NN1). This tissue section comprised typical anatomical features of a healthy lymph node, including the surrounding capsule, multiple primary follicles within the cortex, and the medullary cords and sinuses. The spectral data set collected from the parallel section was again classified by the same neural net and the resulting ANN image is displayed in Figure 3d. This image quite clearly indicates the neural net has not identified any clear regions within the tissue that contain suspicious cells, and has diagnosed the section as being healthy. However, nine red pixels are apparent within the image that were incorrectly classified as being cancerous by the algorithm. These pixels were found at the edges of unrecognised tissue, and may represent regions of very weak spectral features. Nevertheless, the spectral data set classified by the neural net contained 11,000 individual spectra, of which 9 were misclassified.

**Figure 4:**
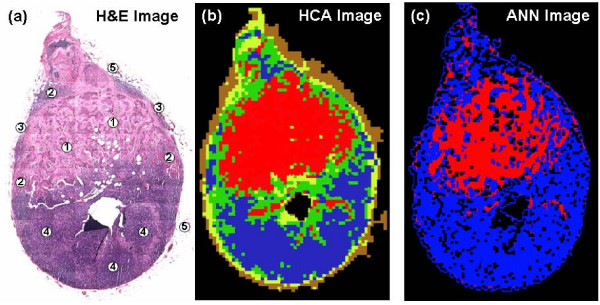
**Deparaffinised tissue analysis using HCA spectroscopic imaging & supervised pattern recognition**. (a) H&E stained image of a positive lymph node PN3. The positive node comprises a large region of cancerous breast tissue (1), macrophages (2), lymphocytes (4), adipose tissue (5), and a thin surrounding capsule (3). (b) HCA spectroscopic image of positive lymph node PN3. The IR imaged area (3.3 mm × 4.9 mm) was mapped using a step size and aperture of 25 μm for a total 25,676 individual IR spectra. The red colour in the image describes areas of cancerous breast tissue. In contrast, the remnant healthy tissues are depicted by the blue (lymphocytes), green (macrophages), brown (adipose tissue) and yellow (capsule) colours respectively. (c) ANN image of positive lymph node PN3. The red colour in the image describes regions correctly classified as cancerous by the analysis. In contrast, the blue colour depicts the remnant healthy tissues that were correctly classified. Black pixels within the image describe spectra that were not able to be classified by the neural net.

The second and directly parallel part of this study was the spectroscopic investigation of deparaffinised lymph nodes tissues. We used the same approach utilised for the frozen sections of tissue and a positive lymph node that contained both breast metastasis and remnant healthy tissues was used for neural net training. The results presented in Figure 4 illustrate the initial unsupervised analysis (HCA) of the recorded spectral data set, and the subsequent neural net training and validation. Figure 4a displays the H&E image captured from the positive lymph node used in this process (labelled as PN3). In contrast to the analysis of frozen tissues, the reflective substrates used for deparaffinised tissues more readily allow the conventional H&E staining of samples. Thus, all subsequent H&E images presented in this paper were collected directly from the section used for spectral data acquisition. After conventional screening by a histopathologist, regions of cancerous invasion, macrophages, capsular tissue, adipose tissue, and cortex tissue containing lymphocytes were identified. By directly comparing this H&E image to cluster maps that were constructed after HCA analysis, a 5-cluster structure provided differentiation of all tissue types present. As can be seen in Figure 4b, the red colour describes the invading metastatic breast cancer, the remnant lymphocytes are a dark blue, macrophages can be identified as green, the thin capsule is yellow, and the surrounding adipose tissue has a brown colouration. The spectra contained in these clusters were then used to train a diagnostic neural net using the same methodology as described previously. Figure 4c displays the ANN image that was constructed after analysing the same spectral data set via this newly trained neural net. The direct comparison of the images acquired from staining (Figure 4a) and HCA spectroscopic imaging (Figure 4b) again shows remarkable agreement between histopathological classification and the spectroscopic method. Regions of the tissue section that were previously identified as being macrophages, cortex, capsule, and adipose tissue were correctly classified by the algorithm as being non-cancerous (blue colour). In contrast, the invading cancerous breast tissue has been correctly classified as being malignant or cancerous in nature (red colour). The amount of additional black pixels is also very small, which again indicates only a few spectra could not be classified by the algorithm.

**Figure 5:**
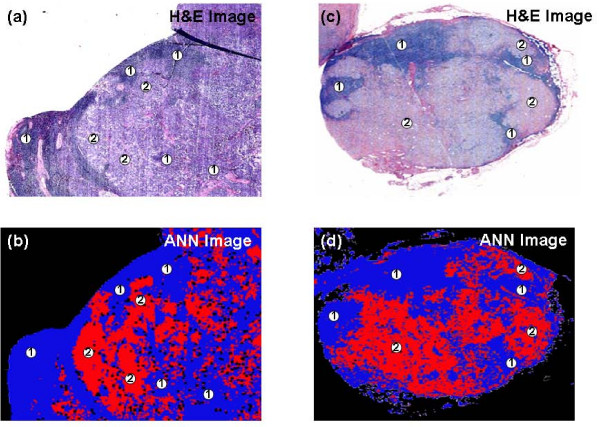
**Deparaffinised tissue analysis using supervised pattern recognition**. (a) H&E stained image of positive lymph node PN4. The IR imaged area (5.2 mm × 4.6 mm) was mapped using a step size and aperture of 25 μm for a total 37,856 individual IR spectra. The positive node comprises large regions of cancerous breast tissue (2) and remnants of healthy nodal tissue (1). (b) ANN image of positive lymph node PN4. The red and blue colours represent the correctly classified cancerous (2) and healthy nodal tissues (1) respectively. Black pixels within the image describe spectra that were not able to be classified by the neural net. (c) H&E stained image of negative lymph node PN5. The IR imaged area (6.5 mm × 6.5 mm) was mapped using a step size and aperture of 25 μm for a total 67,600 individual IR spectra. The positive node comprises both remnant healthy nodal tissue (1) and invading cancerous breast tissue (2). (d) ANN image of negative node PN5. The blue colour is representative of the correctly classified healthy nodal tissue. In contrast, the red colour depicts the regions of cancerous invasion that were correctly classified. Black pixels within the image describe spectra that were not able to be classified by the neural net.

This neural net was subsequently applied to two additional spectral data sets that were recorded from deparaffinised lymph nodes. These spectral diagnoses are presented in Figure 5. Both spectral data sets used to test the algorithm were recorded from positive lymph nodes (labelled PN4 & PN5) that contained clear regions of breast metastatic cancer and remnant healthy cortex tissue. Their corresponding H&E stained photomicrographs are displayed in Figures 5a and 5c respectively. Figures 5b and 5d alternatively display the ANN images that were constructed from spectral classification by use of the neural net. It is clear from direct comparison of these images that regions of cancerous invasion and healthy tissue have been correctly identified by the algorithm and these are described by the red and blue colours respectively. There are only a small number of disparities between histology and spectral diagnosis, which also lie along borders of metastatic invasion where cancerous spectral features are often expressed and identified.

## Conclusion

This paper provides strong evidence that automated diagnosis by means of infrared micro-spectral imaging is possible. By correlating spectral data acquired from unstained tissue, to morphological interpretations made by pathologists of stained tissue, automated algorithms were successfully constructed that can rapidly classify spectral data recorded from frozen and deparaffinised tissue into benign and malignant categories. This would indicate that both intra-operative and more conventional surgical specimens can be diagnosed by this technique, although frozen samples would be preferable since we are assured biochemical integrity is maintained. Present studies are focused toward extending this classification scheme to identify all subtypes of tissue composed with an excised lymph node, which has been successfully accomplished in several different organ models using different methods of supervised analysis [30,37]. However, the application of this technology to solely identify morphological features that are discernable by a pathologist using H&E staining protocols falls short of what is achievable using this technique. Since a change to the molecular composition of a cell most likely occurs before a morphological change, there is a potential to identify abnormalities within tissue at an earlier stage disease. For example, activated lymphocytes that are reacting to an infection, or conversely a breast or metastatic cancer, could display different biochemical characteristics that are identifiable using this technology. Early or "pre-cancerous" stages of abnormality may also be discernable and provide prognostic rather than diagnostic information to a physician. Although the occurrence of such pre-cancerous stages of disease is not directly investigated in this study, the application of HCA spectroscopic imaging may help reveal such tissues. When employing a greater number of clusters to describe the tissue biochemistry than those identified by pathologists as being ideal, distinct bordering regions are often identified between healthy and abnormal tissues that may provide additional sensitivity to identify potentially suspicious cells. Our present focus lies toward the direct investigation and interpretation of such tissues, and the correlation of such intermediate states of disease with other non-morphological interpretations of tissue, such as immunohistochemical stains. The identification of micro features within tissues such as micro-metastases or isolated tumour cells is also vitally important. Recent experiments that have investigated breast micro-metastases within lymph nodes, using a superior pixel resolution of 6.25 μm^2^, have displayed a sensitivity to identify very small regions of abnormal tissue that encompass only a few cancerous cells. Such results are extremely promising, and shall be reported at a later date, but suggest infrared micro-spectral imaging can provide a sensitivity and specificity that rivals current screening protocols. Parallel studies on lymph nodes that display colon metastases have also revealed that cancers from different primary tumours provide distinctly different spectral signatures [44]. These observations have also been reported for similar studies on brain metastatic cancers [45]. Thus poorly differentiated and hard to determine cases of metastatic invasion may additionally be identified by this technique.

The spectroscopic data recorded in this investigation were acquired using instrumentation that employed a small linear array detector system, composed of only 16 detector elements. Thus, acquisition times of spectral data sets from very large lymph nodes was time consuming and in the hour timescale. However, more recently, instrumentation that employ 2^nd ^generation Focal Plane Array (FPA) camera detector systems have become commercially available. These systems can simultaneously record 16,384 infrared spectra over a 700 μm^2 ^area (128 × 128 pixels) with a pixel resolution of 5.5 μm^2^. Consequently, tissue sections that measure 5 mm × 5 mm in size can be spectroscopically imaged in ca. 10 minutes with superior pixel resolution. The rapid and continued development of IR detector array technology and IR instrumentation could feasibly lower this timescale to a few minutes or less.

## Competing interests

The authors declare that they have no competing interests.

## Authors' contributions

BB acquired spectroscopic data from, carried out HCA analysis on, and helped train the ANN for frozen lymph nodes tissues. This work was carried out both at Nottingham University (PhD research) and Northeastern University (Postdoctoral research). BB also drafted the manuscript. MM trained the ANN's for both frozen and de-paraffinised tissues, and was integral for all multivariate analyses at Northeastern University. MR acquired spectroscopic data from, and carried out the HCA analysis upon the de-paraffinised lymph nodes at Northeastern University. MD is principal investigator at Northeastern University, and participated in the study design and co-ordination. JS acquired frozen lymph nodes direct from surgery at Gloucestershire Royal Hospital and was involved in initial spectroscopic data acquisition. NS is principal investigator in the Biophotonics Research Group at Gloucestershire Royal Hospital. He participated in the study design and co-ordination. MG is principal investigator at Nottingham University and participated in the study design and co-ordination. All authors' have read and approved the final manuscript.

## Pre-publication history

The pre-publication history for this paper can be accessed here:


